# Supplementary material for: Molecular Diagnosis of Human Monkeypox Virus during 2022–23 Outbreak: Preliminary Evaluation of Novel Real-Time Qualitative PCR Assays
Source: Microorganisms. 2024 Mar 27;12(4):664. doi: 10.3390/microorganisms12040664 (PMC11052509; doi:10.3390/microorganisms12040664)
Supplement: Supplementary file 1 [file microorganisms-12-00664-s001.zip › microorganisms-2882640-supplementary.pdf]

Supplemental material

Table S1: *Detection of MPXV DNA using four RUO molecular assays*. Positive results were reported as Ct. Abbreviations: Novaplex (Novaplex MPXV Assay); M10 (STANDARD M10 MPX/OPX); RealCycler (RealCycler MONK-UX / MONK-GX v.2); RealStar (RealStar Orthopoxvirus PCR Kit 1.0); weakly positive (+); positive (++). Results with different interpretation from reference molecular procedure were reported in bold red.

				Real-time PCR assays of MPXV			
Sample ID	Specimen Type	Collection date	In-house	Novaplex	M10	RealCycler	*RealStar*
1	Rectal swab	08/07/2022	20 ++	17 ++	19 ++	15 ++	18 ++
2	Hand pustule	08/07/2022	23 ++	20 ++	25 ++	16 ++	22 ++
3	Genital lesion	11/07/2022	18 ++	14 ++	18 ++	11 ++	16 ++
4	Anal lesion	11/07/2022	20 ++	17 ++	18 ++	13 ++	18 ++
5	Vesicle (not specified)	11/07/2022	Negative	Negative	Negative	Negative	Negative
6	Penis lesion	12/07/2022	24 ++	19 ++	21 ++	17 ++	22 ++
7	Penis lesion	12/07/2022	22 ++	16 ++	19 ++	14 ++	20 ++
8	Genital lesion	13/07/2022	22 ++	19 ++	20 ++	15 ++	18 ++
9	Genital lesion	13/07/2022	38 +	37 +	34 +	**30 ++**	34 +
10	Glans lesion	15/07/2022	29 ++	22 ++	24 ++	16 ++	22 ++
11	Penis lesion	15/07/2022	28 ++	19 ++	22 ++	14 ++	20 ++
12	Anal lesion	15/07/2022	25 ++	19 ++	20 ++	14 ++	22 ++
13	Back lesion	15/07/2022	34 +	32 +	32 +	**26 ++**	32 +
14	Lesion (not specified)	15/07/2022	28 ++	23 ++	27 ++	20 ++	25 ++
15	Lesion (not specified)	15/07/2022	28 ++	23 ++	26 ++	19 ++	24 ++
16	Lesion (not specified)	15/07/2022	28 ++	18 ++	22 ++	14 ++	20 ++
17	Foreskin lesion	18/07/2022	Negative	Negative	Negative	Negative	Negative
18	Anal lesion	18/07/2022	Negative	Negative	Negative	Negative	Negative
19	Foreskin lesion	18/07/2022	Negative	Negative	Negative	Negative	Negative
20	Vesicle (not specified)	19/07/2022	Negative	Negative	Negative	Negative	Negative
21	Vesicle (not specified)	20/07/2022	Negative	Negative	Negative	Negative	Negative
22	Vesicle (not specified)	22/07/2022	Negative	Negative	Negative	Negative	Negative
23	Vesicle (not specified)	25/07/2022	Negative	Negative	Negative	Negative	Negative
24	Vesicle (not specified)	26/07/2022	Negative	Negative	Negative	Negative	Negative
25	Pustule (not specified)	26/07/2022	Negative	Negative	Negative	Negative	Negative
26	Lesion (not specified)	29/07/2022	25 ++	20 ++	23 ++	15 ++	20 ++
27	Lesion (not specified)	29/07/2022	23 ++	20 ++	22 ++	14 ++	20 ++
28	Lesion (not specified)	29/07/2022	23 ++	21 ++	23 ++	16 ++	22 ++
29	Lesion (not specified)	29/07/2022	31 +	**25 ++**	**26 ++**	**21 ++**	**26 ++**
30	Anal lesion	02/08/2022	38 +	38 +	35 +	**29 ++**	34 +
31	Lesion (not specified)	02/08/2022	36 +	34 +	33 +	**27 ++**	32 +
32	Skin lesion	03/08/2022	32 +	**25 ++**	**26 ++**	**20 ++**	**26 ++**
33	Lesion (not specified)	03/08/2022	35 +	33 +	33 +	**27 ++**	31 +
34	Anal lesion	05/08/2022	25 ++	23 ++	25 ++	18 ++	22 ++
35	Rectal swab	05/08/2022	18 ++	15 ++	15 ++	11 ++	14 ++
36	Anal lesion	05/08/2022	36 +	37 +	**Negative**	**Negative**	36 +
37	Rectal swab	08/07/2022	20 ++	17 ++	19 ++	15 ++	18 ++
